# Nationwide trends in inpatient shoulder girdle injuries in Germany: a population-based analysis from 2010 to 2023

**DOI:** 10.1186/s40621-026-00668-3

**Published:** 2026-03-06

**Authors:** Houmam Anees, Christian Heiss, Thaqif El Khassawna

**Affiliations:** 1 Department of Trauma, Hand and Reconstructive Surgery, Faculty of Medicine, Justus-Liebig-University of Giessen, 35392 Giessen, Germany; 2 Experimental Trauma Surgery, Faculty of Medicine, Justus-Liebig-University of Giessen, 35392 Giessen, Germany; 3 Biruni University, Istanbul, Turkey; 4 School of Pharmacy, The University of Jordan, Amman, 11942 Jordan

**Keywords:** Shoulder Injuries, Epidemiology, Inpatients, Germany, Nationwide study, COVID-19 pandemic, Hospitalisation rates, Population-based study

## Abstract

**Background:**

Shoulder girdle injuries are common musculoskeletal conditions that frequently require medical attention and may result in substantial functional impairment and healthcare utilisation. While many cases are managed on an outpatient basis, a relevant proportion requires inpatient care. Comprehensive nationwide analyses of inpatient shoulder girdle injuries over extended time periods remain limited, particularly with regard to recent healthcare disruptions such as the COVID-19 pandemic.

**Methods:**

This nationwide, population-based, retrospective study analysed aggregated inpatient hospital discharge data from the German Federal Health Monitoring system (GBE-Bund). Inpatient hospitalisations for shoulder girdle injuries were identified using ICD-10-GM code S43 as the primary discharge diagnosis. The primary analysis covered the continuous annual period from 2010 to 2023, with data from 2000 and 2005 presented as historical reference years. Population-based hospitalisation rates per 100,000 inhabitants were calculated using official population statistics. Analyses were descriptive and included predefined age-stratified subgroup analyses.

**Results:**

Between 2010 and 2023, the annual number of inpatient shoulder girdle injury hospitalisations declined from 23,549 to 17,532 cases. Correspondingly, the population-based hospitalisation rate decreased from 28.8 to 21.0 per 100,000 inhabitants. Hospitalisation rates remained largely stable during the early observation period, followed by a gradual decline and a pronounced reduction in 2020, coinciding with the COVID-19 pandemic. A partial recovery was observed in subsequent years, with rates remaining below pre-pandemic levels. Throughout the study period, inpatient hospitalisations were consistently more frequent in males than in females. Age-stratified analyses showed that inpatient hospitalisations were highest in patients aged 15–44 years, followed by ≥ 65 years and 45–64 years, respectively. The mean length of hospital stay declined steadily from 3.9 days in 2010 to 2.9 days in 2023.

**Conclusions:**

Nationwide inpatient hospitalisations for shoulder girdle injuries in Germany declined overall during the observation period, accompanied by a substantial reduction in length of hospital stay and a persistent male predominance. The marked decline observed during the COVID-19 pandemic and incomplete recovery thereafter highlight evolving patterns in hospital-based trauma care. These findings provide contemporary population-based epidemiological benchmarks and are relevant for healthcare planning and resource allocation.

**Supplementary Information:**

The online version contains supplementary material available at 10.1186/s40621-026-00668-3.

## Introduction

Shoulder girdle injuries are common musculoskeletal conditions that frequently require medical attention and can lead to substantial functional impairment and increased healthcare utilisation [1]. The shoulder girdle comprises the glenohumeral, acromioclavicular, and sternoclavicular joints, whose exceptional range of motion and complex anatomical configuration render this region particularly susceptible to traumatic injury during falls, sports activities, and traffic-related accidents [2, 3]. Injuries affecting this anatomical complex therefore represent a relevant burden for trauma and orthopaedic services, particularly in the inpatient setting.

Epidemiological research on shoulder injuries has largely focused on specific subtypes, most notably glenohumeral dislocations or acromioclavicular joint injuries, and has frequently relied on emergency department data, regional cohorts, or short observation periods [4–6]. While these studies have provided valuable insights into injury mechanisms, age and sex distribution, and trends within selected populations, comprehensive nationwide analyses covering all inpatient shoulder girdle injuries over extended time periods remain limited. In particular, population-based inpatient data reflecting long-term trends in Germany are scarce.

The COVID-19 pandemic substantially affected healthcare utilisation worldwide, leading to marked reductions in hospital admissions across a wide range of medical and surgical conditions [7–9]. Injury-related hospitalisations were also influenced by pandemic-related behavioural changes, mobility restrictions, and altered healthcare-seeking behaviour [10]. However, the specific impact of the pandemic on inpatient hospitalisations for shoulder girdle injuries at a national level has not yet been systematically investigated.

Nationwide administrative health databases provide an opportunity to assess long-term injury trends across entire populations with minimal selection bias. In Germany, the Federal Health Monitoring system (GBE-Bund) offers publicly accessible, standardised inpatient data coded according to the International Classification of Diseases (ICD), enabling robust temporal comparisons across calendar years [11]. Analyses of such data allow the identification of secular trends, sex-specific patterns, and potential disruptions in healthcare utilisation.

Over recent years, healthcare systems in Europe have increasingly shifted selected surgical and musculoskeletal treatments from inpatient to outpatient settings. This transition has been driven by advances in surgical techniques, perioperative management, and health policy initiatives aimed at improving efficiency and reducing healthcare costs while maintaining quality of care [12]. In addition, economic analyses comparing inpatient and outpatient surgical procedures have demonstrated the growing relevance of ambulatory care pathways and their potential impact on overall healthcare resource utilisation [13].

The aim of this study was therefore to examine nationwide temporal trends in inpatient shoulder girdle injuries in Germany over a prolonged observation period from 2010 to 2023. By analysing population-based hospitalisation rates and sex distribution, this study seeks to provide contemporary epidemiological benchmarks and to characterise changes in inpatient care patterns before, during, and after the COVID-19 pandemic.

## Methods

### Study design and data source

This study is a nationwide, population-based, retrospective analysis of inpatient hospitalisations for shoulder girdle injuries in Germany. Aggregated administrative hospital discharge data were obtained from the German Federal Health Monitoring system (GBE-Bund), which provides publicly accessible, standardised national inpatient statistics [11]. Population data were retrieved from the Federal Statistical Office of Germany (Destatis) [14]. Diagnoses are coded according to the German modification of the International Classification of Diseases, 10th Revision (ICD-10-GM), which is updated annually by the Federal Institute for Drugs and Medical Devices (BfArM).

The dataset represents discharge-level case data rather than individual patient-level data. Consequently, repeated hospitalisations of the same patient cannot be identified and may be included more than once. The database captures acute inpatient hospitalisations within the German DRG-based hospital system. Outpatient care, emergency department contacts without admission, and inpatient rehabilitation episodes are not included [11]. In Germany, most acute traumatic shoulder injuries requiring inpatient treatment are admitted via emergency departments, although elective admissions may occur in selected cases [11].

### Case definition

Inpatient cases were identified using ICD-10-GM code S43 (“Luxation, sprain and strain of joints and ligaments of the shoulder girdle”) recorded as the main discharge diagnosis. This code group includes traumatic injuries of the glenohumeral, acromioclavicular, and sternoclavicular joints. The S43 category includes the following ICD-10-GM subcodes: S43.0–S43.9 (see Supplementary Table S1). All inpatient hospitalisations with ICD-10-GM S43 as the primary diagnosis were included. No additional ICD subcodes were analysed.

The main diagnostic category was selected to ensure consistent case identification across the long observation period and to minimise bias related to temporal changes in subcode usage.

### Study population and observation period

All inpatient shoulder girdle injury cases recorded nationwide were included without age restriction. The primary analysis covered the continuous annual time series from 2010 to 2023, for which complete nationwide data were available.

Data from 2000 and 2005 were additionally extracted and are presented as historical reference points. These years represent the only earlier nationally available inpatient datasets accessible via the German Federal Health Monitoring system (GBE-Bund) prior to 2010 and were therefore included solely for descriptive contextual comparison. Owing to the absence of continuous annual data between these time points, they were not included in the longitudinal trend analysis.

### Population data

Annual population figures were obtained from official statistics provided by the German Federal Statistical Office (Destatis) via the GBE-Bund system [14]. Population counts refer to the resident population at 31 December of each calendar year and were used as denominators for calculating population-based hospitalisation rates.

### Outcome measures

Primary outcomes were:

- Annual number of inpatient shoulder girdle injury hospitalisations.
- Population-based hospitalisation rates per 100,000 inhabitants.

Secondary descriptive variables included:

- Sex distribution (male, female).
- Mean length of hospital stay (days).

Age-stratified inpatient case numbers were additionally extracted from publicly available GBE-Bund age-group tables and analysed descriptively. These data were used to generate age-group trend analyses (< 15, 15–44, 45–64, ≥ 65 years) and to construct Supplementary Table S2.

However, individual-level linkage of age with sex, injury severity, treatment modality, or comorbidities was not possible. Injury mechanism, injury severity (e.g., Abbreviated Injury Scale), specific trauma mechanism, surgical treatment, and inpatient rehabilitation episodes were not available.

### Statistical analysis

Hospitalisation rates were calculated by dividing the annual number of inpatient S43 cases by the corresponding total population and multiplying by 100,000. Rates are presented per 100,000 inhabitants. All analyses were descriptive. Because the dataset represents complete nationwide aggregated data rather than a sample, inferential statistical testing was not performed. Measures of variability, including confidence intervals for length of hospital stay, could not be calculated because the publicly available GBE-Bund data provide aggregated mean values only and do not include information on variance or individual-level observations. Results are presented using tables and figures. Age-group analyses were performed descriptively using absolute annual case numbers.

## Results

### Nationwide inpatient case numbers and hospitalisation rates

Between 2010 and 2023, the annual number of inpatient hospitalisations for shoulder girdle injuries (ICD-10-GM S43) in Germany declined overall, despite notable temporal fluctuations (Table 1). Total inpatient case numbers decreased from 23,549 cases in 2010 to 17,532 cases in 2023. Correspondingly, the population-based hospitalisation rate declined from 28.8 per 100,000 inhabitants in 2010 to 21.0 per 100,000 inhabitants in 2023. The ICD-10-GM subcodes included within the S43 category are listed in Supplementary Table S1.

**Table 1:** Nationwide annual number of inpatient hospitalisations for shoulder girdle injuries (ICD-10-GM S43) in Germany and corresponding population-based hospitalisation rates per 100,000 inhabitants

Year	Total inpatient S43 cases (all ages)	Male	Female	Length of stay in Days (mean)	Population	Hospitalisation rate per 100,000*
2023	17,532	12,825	4707	2.9	83,456,045	21.0
2022	19,899	14,807	5092	2.9	83,118,501	23.9
2021	18,831	13,405	5426	3.1	83,237,124	22.6
2020	20,728	14,819	5909	3.1	83,155,031	24.9
2019	22,955	16,471	6484	3.3	83,166,711	27.6
2018	23,413	16,889	6524	3.4	83,019,213	28.2
2017	23,553	16,585	6968	3.4	82,792,351	28.4
2016	23,773	16,780	6993	3.4	82,521,653	28.8
2015	23,991	17,137	6854	3.4	82,175,684	29.2
2014	23,994	16,959	7035	3.6	81,197,537	29.6
2013	24,123	16,816	7307	3.7	80,767,463	29.9
2012	24,342	17,123	7219	3.7	80,523,746	30.2
2011	23,748	16,790	6958	3.8	80,327,900	29.6
2010	23,549	16,474	7075	3.9	81,751,602	28.8
-	-				-	-
2005^†^	21,720	15,417	6303	4.7	82,437,995	26.3
2000^†^	26,133	18,527	7606	5.9	82,259,540	31.8

Rates were calculated by dividing the total number of inpatient S43 cases per calendar year by the corresponding total population and multiplying by 100,000. Sex-specific case numbers are shown descriptively

The primary analysis covers the years 2010–2023, for which complete annual nationwide data were available

Data for the years 2000 and 2005 are presented as historical reference points and are not part of the continuous annual time series

*Hospitalisation rates are rounded to one decimal place

^†^Historical reference years

**Fig. 1:**
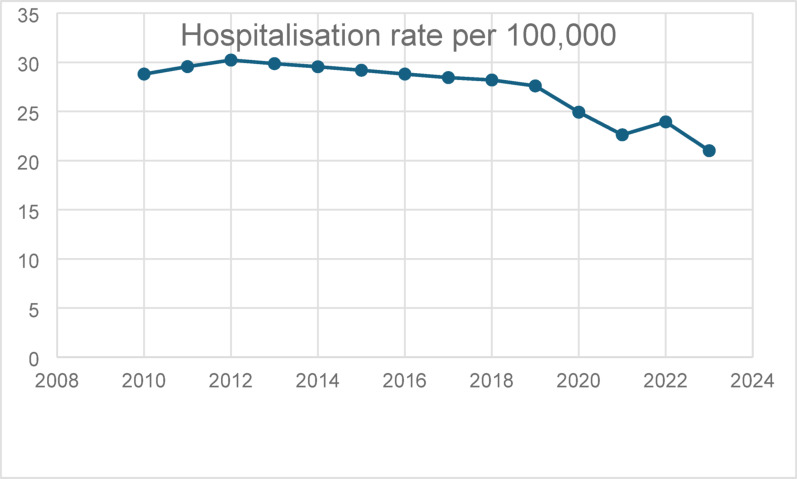
Annual population-based hospitalisation rates for shoulder girdle injuries (ICD-10-GM S43) in Germany between 2010 and 2023, expressed as cases per 100,000 inhabitants. Rates were calculated using nationwide inpatient case numbers and corresponding annual population data. A pronounced decline in hospitalisation rates is observed during the COVID-19 pandemic period (2020–2021), followed by a partial recovery thereafter

Hospitalisation rates increased gradually between 2010 and 2012, followed by a plateau and a gradual decline until 2019. A pronounced reduction was observed in 2020, coinciding with the COVID-19 pandemic, after which rates partially rebounded but remained below pre-pandemic levels through 2023 (Fig. 1).

### Sex-specific trends

Across all years analysed, inpatient shoulder girdle injury hospitalisations were more frequent in males than in females (Table 1). The proportion of male and female cases remained relatively stable throughout the observation period, with males consistently accounting for approximately 70–75% of cases annually. In 2023, males accounted for 12,825 cases (73.1%), compared with 4,707 cases (26.9%) among females.

**Fig. 2:**
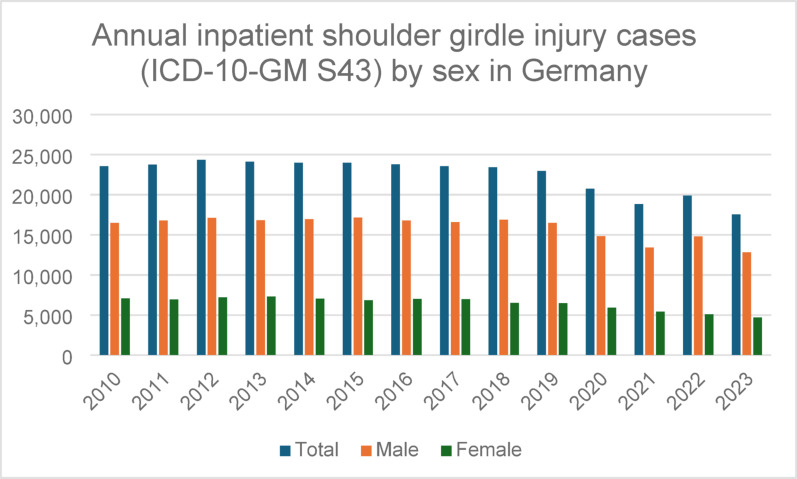
Annual number of inpatient hospitalisations for shoulder girdle injuries (ICD-10-GM S43) in Germany stratified by sex between 2010 and 2023. Male patients consistently accounted for the majority of hospitalised cases throughout the study period, with a stable sex distribution over time

Both sexes exhibited a similar temporal pattern, with declining inpatient case numbers over time and a marked reduction in 2020. However, absolute case numbers remained consistently higher among males throughout the observation period (Fig. 2).

### Age-specific trends

**Fig. 3:**
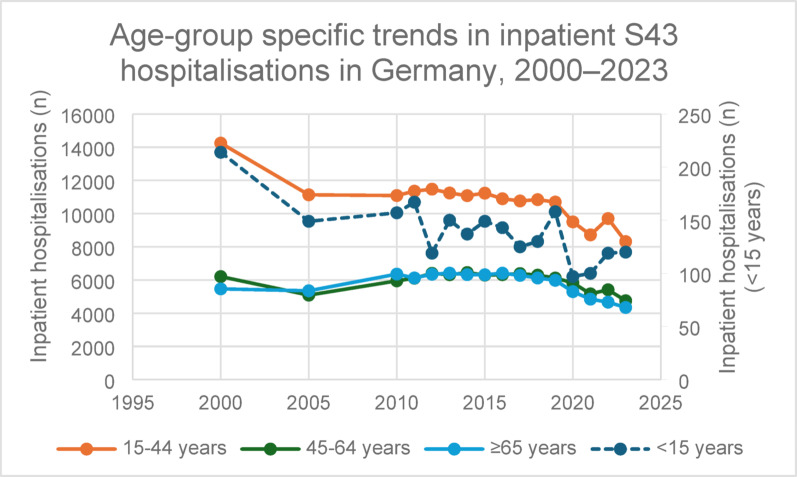
Age-group specific trends in nationwide inpatient hospitalisations for shoulder girdle injuries (ICD-10-GM S43) in Germany, 2000–2023. Annual inpatient case numbers are shown stratified into four age groups: <15 years, 15–44 years, 45–64 years, and ≥ 65 years. Data were obtained from the German Federal Health Monitoring system (GBE-Bund). The < 15 years group is displayed on a secondary y-axis to allow visual comparison with adult age groups. Overall, inpatient hospitalisations declined across adult age groups over time, with a marked reduction during the COVID-19 pandemic period (2020–2021) and incomplete recovery thereafter

Age-group stratified analyses demonstrated that inpatient hospitalisations were highest in the 15–44 year age group throughout the observation period, followed by patients aged ≥ 65 years and 45–64 years, respectively (Fig. 3). All adult age groups showed a decline over time, with a pronounced decrease during the COVID-19 pandemic period and only partial recovery thereafter. Detailed age-stratified annual case numbers are provided in Supplementary Table S2. These data reflect acute inpatient hospitalisations only and do not include inpatient rehabilitation episodes.

### Length of hospital stay

The mean length of hospital stay decreased steadily over the study period (Table 1). The mean length of stay declined from 3.9 days in 2010 to 2.9 days in 2023. This downward trend was observed consistently across calendar years, including during the COVID-19 pandemic period.

### Historical reference years

For contextual comparison, data from the years 2000 and 2005 are shown as historical reference points (Table 1). These years demonstrated higher inpatient case numbers, hospitalisation rates, and longer mean lengths of stay compared with the later observation period but were not included in the continuous annual trend analysis. They represent isolated earlier nationwide data points available via GBE-Bund and are presented solely to provide historical context rather than to infer temporal trends.

### Summary of key findings

Overall, nationwide inpatient hospitalisations for shoulder girdle injuries in Germany declined over the study period, with a marked reduction during the COVID-19 pandemic and incomplete recovery thereafter. Hospitalisation rates and mean length of stay decreased substantially over time, while a persistent male predominance was observed throughout the observation period.

## Discussion

This nationwide population-based analysis demonstrates a largely stable pattern of inpatient hospitalisations for shoulder girdle injuries in Germany until approximately 2019, followed by a subsequent decline, most pronounced during the COVID-19 pandemic period, accompanied by a continuous reduction in length of hospital stay and a stable male predominance. Age-stratified analyses further demonstrated that the highest inpatient burden was consistently observed in the 15–44 year age group, followed by ≥ 65 years and 45–64 years, respectively. These combined findings likely reflect a combination of epidemiological trends, demographic structure, and structural changes in healthcare delivery rather than a simple linear decline in injury burden and provide robust epidemiological benchmarks for inpatient shoulder girdle injuries at the national level.

### Long-term decline in inpatient hospitalisations

The observed reduction in inpatient shoulder girdle injury hospitalisations from 2010 to 2023 suggests changes in inpatient care utilisation and management pathways rather than necessarily a true decrease in overall injury occurrence. Advances in diagnostic imaging, surgical techniques, and conservative treatment strategies, as well as the increasing availability of outpatient and ambulatory care pathways, may have reduced the need for inpatient admission. Similar trends have been reported for other musculoskeletal and trauma-related conditions, indicating a broader transformation of inpatient care delivery [15]. The age-stratified results suggest that this shift may be particularly relevant for working-age adults (15–44 years), who represent the largest proportion of inpatient cases and may benefit most from expanding outpatient treatment pathways and early functional rehabilitation strategies.

Importantly, declining inpatient rates should not be interpreted as a proxy for declining injury incidence. Emergency department–based studies have reported stable or increasing presentations for shoulder injuries in selected demographic or activity-based populations, such as athletes or younger trauma cohorts, underscoring that a growing proportion of cases may now be managed without hospital admission [4, 6]. Nationwide inpatient data therefore capture only the more severe end of the clinical spectrum and primarily reflect changes in admission thresholds and care organisation. In addition, structural health system factors, including the expansion of outpatient surgical capacity and increasing efficiency pressures in inpatient care, may have contributed to declining hospitalisation rates over time [12, 15].

### Impact of the COVID-19 pandemic

A pronounced reduction in inpatient shoulder girdle injury hospitalisations was observed in 2020, coinciding with the COVID-19 pandemic. This decline was observed across all adult age groups, suggesting system-wide changes in exposure risk and healthcare utilisation rather than age-specific effects. This finding aligns with reports from Germany and other countries describing substantial declines in hospital admissions during lockdown periods, including trauma-related admissions [7, 8, 10]. In addition, analyses from German trauma systems and hospital-based studies have demonstrated marked reductions in trauma case volumes during the first lockdown phase, supporting the interpretation that pandemic-related restrictions substantially influenced injury incidence and hospital utilisation patterns [16].

Reduced mobility, suspension of organised sports, decreased traffic volume, and altered risk exposure likely contributed to fewer injury events. These behavioural and environmental changes are supported by German data demonstrating reductions in trauma caseloads and shifts in trauma care utilisation during lockdown periods, as well as reductions in prehospital emergency utilisation [17, 18]. In parallel, changes in healthcare-seeking behaviour and prioritisation of hospital resources may have further limited admissions, particularly for injuries that could be managed conservatively or in outpatient settings.

Notably, inpatient hospitalisation rates did not fully return to pre-pandemic levels in subsequent years. This incomplete recovery suggests that some pandemic-related changes, such as increased reliance on outpatient management pathways or revised admission practices, may have persisted beyond the acute phase of the pandemic. Whether these patterns represent permanent shifts in trauma care or delayed catch-up effects warrants continued surveillance. Future analyses integrating inpatient and outpatient data sources will be important to better understand the long-term impact of the pandemic on injury epidemiology and trauma care pathways.

### Sex-specific patterns

Throughout the study period, inpatient shoulder girdle injuries were consistently more frequent in males, accounting for approximately three-quarters of hospitalised cases. This stable male predominance mirrors findings from prior epidemiological studies and likely reflects sex-specific differences in exposure to occupational hazards, sports participation, and high-energy trauma [5, 6]. The persistence of this pattern despite declining overall hospitalisation rates suggests that underlying risk structures have remained largely unchanged.

### Length of hospital stay

The steady decline in mean length of hospital stay over time represents an additional key finding. Shorter hospital stays likely reflect improvements in perioperative management, early mobilisation protocols, enhanced recovery strategies, and greater efficiency in inpatient care delivery. At the same time, declining hospitalisation rates may indicate that only more severe cases are admitted, potentially influencing inpatient case mix and requiring careful interpretation of length-of-stay trends. This trend was observed consistently across the study period, including during the COVID-19 pandemic period, and highlights ongoing optimisation of hospital resource utilisation.

### Strengths and limitations

A major strength of this study is the use of complete nationwide inpatient data with standardised ICD-10-GM coding, minimising selection bias and enabling robust temporal comparisons [11]. The long observation period allows the assessment of secular trends as well as pandemic-related disruptions.

Several limitations must be acknowledged. First, reliance on administrative data may be affected by coding inaccuracies or changes in coding practices over time, which may influence observed temporal trends independent of true epidemiological changes. Second, the analysis is restricted to inpatient hospitalisations and does not capture outpatient care, emergency department visits without admission, inpatient rehabilitation admissions, or injury severity. Importantly, the GBE-Bund inpatient discharge statistics reflect acute-care hospitalisations within the DRG-based hospital system and do not include stays in rehabilitation facilities, even when rehabilitation is delivered in an inpatient setting. This limitation may lead to underestimation of the total burden of shoulder girdle injuries and may partially explain the observed decline in inpatient hospitalisations if an increasing proportion of cases are managed in outpatient settings or transferred to dedicated rehabilitation facilities after acute care. Third, information on injury mechanism, injury severity, treatment modality, and comorbidities was not available. The absence of these variables limits risk stratification and prevents adjustment for potential confounders that may influence hospitalisation rates and length of stay. Fourth, the use of discharge-level aggregated data prevents identification of repeated admissions of the same patient, which may result in overestimation of case numbers if individual patients experienced multiple hospital admissions during the study period.

Finally, the descriptive design precludes causal inference regarding the drivers of observed trends. Accordingly, the observed associations should be interpreted as population-level temporal patterns rather than direct evidence of causal relationships between healthcare system changes and hospitalisation rates. Although age-stratified case numbers were available, age-specific population denominators were not analysed, preventing calculation of age-standardised hospitalisation rates. Therefore, age analyses should be interpreted as case burden rather than true age-specific incidence.

### Implications for injury epidemiology and healthcare planning

Despite these limitations, the present study contributes important population-level evidence on the evolving inpatient burden of shoulder girdle injuries. The findings underscore the necessity of integrating inpatient, outpatient, and rehabilitation care data sources to fully characterise injury epidemiology and inform healthcare planning. Where possible, linkage of acute-care hospitalisation data with inpatient rehabilitation facility statistics would allow a more complete assessment of system-wide resource use following shoulder girdle injuries in Germany. The high inpatient burden in the 15–44 year age group highlights the socioeconomic relevance of shoulder girdle injuries, particularly with regard to work-related disability and productivity loss. Continued monitoring of inpatient trends remains essential for anticipating resource needs, particularly in the context of demographic change and evolving care models.

## Conclusions

Nationwide inpatient hospitalisations for shoulder girdle injuries in Germany have declined overall across major age groups, particularly in adult populations over the observation period, accompanied by a substantial reduction in mean length of hospital stay and a persistent male predominance. A marked decrease in hospitalisations during the COVID-19 pandemic was observed, with only partial recovery thereafter. These findings provide contemporary population-based benchmarks for inpatient shoulder girdle injuries and highlight evolving patterns of hospital-based trauma care, which are relevant for healthcare planning and resource allocation.

## Supplementary Information

Supplementary Material 1

Supplementary Material 2

## Data Availability

All data analysed in this study are publicly available via the German Federal Health Monitoring system (GBE-Bund) . The aggregated data used for analysis are included in this article.
