# Supplementary material for: Nationwide trends in inpatient shoulder girdle injuries in Germany: a population-based analysis from 2010 to 2023
Source: Inj Epidemiol. 2026 Mar 6;13:24. doi: 10.1186/s40621-026-00668-3 (PMC13047791; doi:10.1186/s40621-026-00668-3)
Supplement: Supplementary file 1 — Supplementary Material 1 [file 40621_2026_668_MOESM1_ESM.docx]

**Nationwide trends in inpatient shoulder girdle injuries in Germany: a population-based analysis from 2010 to 2023**

Houmam Anees^1,2^*****, Christian Heiß^1,2,3^ and Thaqif El Khassawna^2,4^

^1^Department of Trauma, Hand and Reconstructive Surgery, Faculty of Medicine, Justus-Liebig-University of Giessen, 35392 Giessen, Germany

^2^Experimental Trauma Surgery, Faculty of Medicine, Justus-Liebig-University of Giessen, 35392 Giessen, Germany

^3^Biruni University, Istanbul, Türkiye

^4^School of Pharmacy, The University of Jordan, Amman 11942, Jordan

***Corresponding Author:** [Houmam.Anees@chiru.med.uni-giessen.de](mailto:Houmam.Anees@chiru.med.uni-giessen.de) (H. Anees).

**Supplementary Table S2. Age-stratified inpatient S43 case numbers, Germany 2000–2023**

| **Year** | **2023** | **2022** | **2021** | **2020** | **2019** | **2018** | **2017** | **2016** | **2015** | **2014** | **2013** | **2012** | **2011** | **2010** | **2005** | **2000** |
| --- | --- | --- | --- | --- | --- | --- | --- | --- | --- | --- | --- | --- | --- | --- | --- | --- |
| **All age groups** | **17532** | **19899** | **18831** | **20728** | **22955** | **23413** | **23553** | **23773** | **23991** | **23994** | **24123** | **24342** | **23748** | **23549** | **21720** | **26133** |
| **Under 65 years** | **13186** | **15234** | **13985** | **15428** | **16973** | **17265** | **17275** | **17361** | **17676** | **17668** | **17711** | **17995** | **17625** | **17194** | **16370** | **20668** |
| **Under 15 years** | **120** | **119** | **100** | **97** | **158** | **130** | **125** | **143** | **149** | **137** | **150** | **119** | **167** | **157** | **149** | **214** |
| **Under 5 years** | **6** | **2** | **5** | **8** | **5** | **9** | **7** | **11** | **7** | **9** | **4** | **11** | **6** | **8** | **9** | **10** |
| **Under 1 year** | **1** | **1** | **3** | **4** | **2** | **3** | **2** | **3** | **5** | **3** | **1** | **4** | **3** | **5** | **3** | **5** |
| **1 year to under 5 years** | **5** | **1** | **2** | **4** | **3** | **6** | **5** | **8** | **2** | **6** | **3** | **7** | **3** | **3** | **6** | **5** |
| **5 to under 15 years** | **114** | **117** | **95** | **89** | **153** | **121** | **118** | **132** | **142** | **128** | **146** | **108** | **161** | **149** | **140** | **204** |
| **5 to under 10 years** | **10** | **4** | **2** | **2** | **6** | **7** | **12** | **10** | **7** | **8** | **11** | **10** | **3** | **5** | **6** | **21** |
| **10 to under 15 years** | **104** | **113** | **93** | **87** | **147** | **114** | **106** | **122** | **135** | **120** | **135** | **98** | **158** | **144** | **134** | **183** |
| **15 to under 45 years** | **8315** | **9702** | **8719** | **9501** | **10696** | **10842** | **10763** | **10896** | **11232** | **11081** | **11242** | **11473** | **11352** | **11088** | **11132** | **14247** |
| **15 to under 25 years** | **2808** | **3238** | **2881** | **3082** | **3737** | **3789** | **3767** | **3879** | **3982** | **4009** | **4051** | **4220** | **4221** | **4060** | **3623** | **4050** |
| **15 to under 20 years** | **1062** | **1291** | **1042** | **1153** | **1445** | **1466** | **1400** | **1503** | **1441** | **1434** | **1441** | **1475** | **1482** | **1495** | **1497** | **1715** |
| **20 to under 25 years** | **1746** | **1947** | **1839** | **1929** | **2292** | **2323** | **2367** | **2376** | **2541** | **2575** | **2610** | **2745** | **2739** | **2565** | **2126** | **2335** |
| **25 to under 45 years** | **5507** | **6464** | **5838** | **6419** | **6959** | **7053** | **6996** | **7017** | **7250** | **7072** | **7191** | **7253** | **7131** | **7028** | **7509** | **10197** |
| **25 to under 35 years** | **3135** | **3687** | **3314** | **3795** | **4120** | **4178** | **4220** | **4225** | **4341** | **4101** | **4116** | **4057** | **3805** | **3573** | **3397** | **5340** |
| **25 to under 30 years** | **1662** | **1970** | **1693** | **1994** | **2202** | **2342** | **2397** | **2432** | **2546** | **2362** | **2370** | **2331** | **2178** | **1977** | **1769** | **2494** |
| **30 to under 35 years** | **1473** | **1717** | **1621** | **1801** | **1918** | **1836** | **1823** | **1793** | **1795** | **1739** | **1746** | **1726** | **1627** | **1596** | **1628** | **2846** |
| **35 to under 45 years** | **2372** | **2777** | **2524** | **2624** | **2839** | **2875** | **2776** | **2792** | **2909** | **2971** | **3075** | **3196** | **3326** | **3455** | **4112** | **4857** |
| **35 to under 40 years** | **1261** | **1393** | **1313** | **1367** | **1492** | **1532** | **1485** | **1475** | **1470** | **1397** | **1349** | **1396** | **1432** | **1522** | **2060** | **2737** |
| **40 to under 45 years** | **1111** | **1384** | **1211** | **1257** | **1347** | **1343** | **1291** | **1317** | **1439** | **1574** | **1726** | **1800** | **1894** | **1933** | **2052** | **2120** |
| **45 to under 65 years** | **4751** | **5413** | **5166** | **5830** | **6119** | **6293** | **6387** | **6322** | **6295** | **6450** | **6319** | **6403** | **6106** | **5949** | **5089** | **6207** |
| **45 to under 55 years** | **2256** | **2599** | **2619** | **3046** | **3211** | **3441** | **3568** | **3613** | **3681** | **3780** | **3799** | **3826** | **3775** | **3563** | **2929** | **3041** |
| **45 to under 50 years** | **1051** | **1167** | **1158** | **1352** | **1488** | **1564** | **1661** | **1793** | **1896** | **1988** | **1998** | **2084** | **2098** | **1945** | **1631** | **1641** |
| **50 to under 55 years** | **1205** | **1432** | **1461** | **1694** | **1723** | **1877** | **1907** | **1820** | **1785** | **1792** | **1801** | **1742** | **1677** | **1618** | **1298** | **1400** |
| **55 to under 65 years** | **2495** | **2814** | **2547** | **2784** | **2908** | **2852** | **2819** | **2709** | **2614** | **2670** | **2520** | **2577** | **2331** | **2386** | **2160** | **3166** |
| **55 to under 60 years** | **1340** | **1565** | **1354** | **1573** | **1688** | **1593** | **1537** | **1484** | **1437** | **1464** | **1323** | **1305** | **1229** | **1299** | **1103** | **1582** |
| **60 to under 65 years** | **1155** | **1249** | **1193** | **1211** | **1220** | **1259** | **1282** | **1225** | **1177** | **1206** | **1197** | **1272** | **1102** | **1087** | **1057** | **1584** |
| **65 years and older** | **4346** | **4665** | **4846** | **5300** | **5982** | **6122** | **6278** | **6412** | **6315** | **6326** | **6412** | **6347** | **6123** | **6355** | **5350** | **5465** |
| **65 to under 75 years** | **1691** | **1733** | **1763** | **1836** | **2052** | **2106** | **2103** | **2156** | **2266** | **2346** | **2460** | **2576** | **2544** | **2836** | **2395** | **2460** |
| **65 to under 70 years** | **902** | **933** | **911** | **966** | **1155** | **1117** | **1082** | **1097** | **1074** | **1029** | **1028** | **1062** | **1025** | **1213** | **1311** | **1263** |
| **70 to under 75 years** | **789** | **800** | **852** | **870** | **897** | **989** | **1021** | **1059** | **1192** | **1317** | **1432** | **1514** | **1519** | **1623** | **1084** | **1197** |
| **75 years and older** | **2655** | **2932** | **3083** | **3464** | **3930** | **4016** | **4175** | **4256** | **4049** | **3980** | **3952** | **3771** | **3579** | **3519** | **2955** | **3005** |
| **75 to under 85 years** | **1530** | **1772** | **1934** | **2221** | **2596** | **2717** | **2834** | **2880** | **2780** | **2698** | **2619** | **2544** | **2382** | **2397** | **2121** | **2005** |
| **75 to under 80 years** | **660** | **778** | **839** | **1018** | **1261** | **1421** | **1528** | **1628** | **1583** | **1577** | **1479** | **1380** | **1273** | **1259** | **1085** | **1256** |
| **80 to under 85 years** | **870** | **994** | **1095** | **1203** | **1335** | **1296** | **1306** | **1252** | **1197** | **1121** | **1140** | **1164** | **1109** | **1138** | **1036** | **749** |
| **85 years and older** | **1125** | **1160** | **1149** | **1243** | **1334** | **1299** | **1341** | **1376** | **1269** | **1282** | **1333** | **1227** | **1197** | **1122** | **834** | **1000** |
| **85 to under 90 years** | **771** | **717** | **728** | **788** | **833** | **795** | **821** | **889** | **827** | **853** | **884** | **840** | **829** | **800** | **487** | **664** |
| **90 years and older** | **354** | **443** | **421** | **455** | **501** | **504** | **520** | **487** | **442** | **429** | **449** | **387** | **368** | **322** | **347** | **336** |
| **90 to under 95 years** | **276** | **347** | **332** | **360** | **389** | **413** | **428** | **400** | **380** | **372** | **376** | **312** | **279** | **224** | **282** | **282** |
| **95 years and older** | **78** | **96** | **89** | **95** | **112** | **91** | **92** | **87** | **62** | **57** | **73** | **75** | **89** | **98** | **65** | **54** |
| **95 to under 100 years** | **73** | **85** | **84** | **90** | **107** | **86** | **85** | **79** | **57** | **45** | **63** | **67** | **79** | **90** | **0** | **0** |
| **100 years and older** | **5** | **11** | **5** | **5** | **5** | **5** | **7** | **8** | **5** | **12** | **10** | **8** | **10** | **8** | **0** | **0** |

**Supp. Table 2:** Age-stratified annual inpatient case numbers for shoulder girdle injuries (ICD-10-GM S43) in Germany, 2000–2023. Data were obtained from the German Federal Health Monitoring system (GBE-Bund) and represent nationwide aggregated discharge-level inpatient cases. Age groups are presented hierarchically according to the original GBE-Bund classification. Values represent absolute annual case numbers. Data include acute inpatient hospitalisations only and do not capture outpatient care, emergency department visits without admission, or inpatient rehabilitation episodes. Repeated admissions of the same patient cannot be identified due to the aggregated data structure.
