# Supplementary material for: Nationwide trends in inpatient shoulder girdle injuries in Germany: a population-based analysis from 2010 to 2023
Source: Inj Epidemiol. 2026 Mar 6;13:24. doi: 10.1186/s40621-026-00668-3 (PMC13047791; doi:10.1186/s40621-026-00668-3)
Supplement: Supplementary file 2 — Supplementary Material 2 [file 40621_2026_668_MOESM2_ESM.docx]

**Nationwide trends in inpatient shoulder girdle injuries in Germany: a population-based analysis from 2010 to 2023**

Houmam Anees^1,2^*****, Christian Heiß^1,2,3^ and Thaqif El Khassawna^2,4^

^1^Department of Trauma, Hand and Reconstructive Surgery, Faculty of Medicine, Justus-Liebig-University of Giessen, 35392 Giessen, Germany

^2^Experimental Trauma Surgery, Faculty of Medicine, Justus-Liebig-University of Giessen, 35392 Giessen, Germany

^3^Biruni University, Istanbul, Türkiye

^4^School of Pharmacy, The University of Jordan, Amman 11942, Jordan

***Corresponding Author:** [Houmam.Anees@chiru.med.uni-giessen.de](mailto:Houmam.Anees@chiru.med.uni-giessen.de) (H. Anees).

**Supplementary Table S1. ICD-10-GM codes included in the S43 diagnostic category**

| No. | ICD-10-GM Code | Description |
| --- | --- | --- |
| 1 | S43.0 | Dislocation of shoulder joint (glenohumeral joint) |
| 2 | S43.1 | Dislocation of acromioclavicular joint |
| 3 | S43.2 | Dislocation of sternoclavicular joint |
| 4 | S43.3 | Ligament injury of shoulder girdle |
| 5 | S43.4 | Sprain of shoulder joint |
| 6 | S43.5 | Sprain of acromioclavicular joint |
| 7 | S43.6 | Sprain of sternoclavicular joint |
| 8 | S43.7 | Multiple injuries of shoulder girdle |
| 9 | S43.8 | Other specified injuries of shoulder girdle |
| 10 | S43.9 | Unspecified injury of shoulder girdle |

**Supp. Table 1:** ICD-10-GM = German modification of the International Classification of Diseases, 10th Revision. The S43 category comprises traumatic dislocations, sprains, and ligament injuries affecting the shoulder girdle region, including the glenohumeral, acromioclavicular, and sternoclavicular joints. All codes listed above were included within the case definition for inpatient shoulder girdle injuries in this study.
